# *In Vitro* Antiviral Activity of Potential Medicinal Plant Extracts Against Dengue and Chikungunya Viruses

**DOI:** 10.3389/fcimb.2022.866452

**Published:** 2022-04-07

**Authors:** Kalichamy Alagarasu, Poonam Patil, Meenakshi Kaushik, Deepika Chowdhury, Rajesh K. Joshi, Harsha V. Hegde, Mahadeo B. Kakade, Sugeerappa Laxmanappa Hoti, Sarah Cherian, Deepti Parashar

**Affiliations:** ^1^ Dengue and Chikungunya Group, Indian Council of Medical Research (ICMR)-National Institute of Virology, Pune, India; ^2^ Department of Natural Product Chemistry, Indian Council of Medical Research (ICMR)-National Institute of Traditional Medicine, Belagavi, India; ^3^ Department of Ethnomedicine, Indian Council of Medical Research (ICMR)-National Institute of Traditional Medicine, Belagavi, India; ^4^ Ex-Director, Indian Council of Medical Research (ICMR)-National Institute of Traditional Medicine, Belagavi, India

**Keywords:** dengue virus, chikungunya virus, plant extracts, phytopharmaceuticals, antivirals

## Abstract

Dengue and chikungunya are two important mosquito-borne infections which are known to occur extensively in tropical and subtropical areas. Presently, there is no treatment for these viral diseases. *In vitro* antiviral screening of 25 extracts prepared from the plants of *Vitex negundo*, *Plumeria alba*, *Ancistrocladus heyneanus*, *Bacopa monnieri*, *Anacardium occidentale*, *Cucurbita maxima*, *Simarouba glauca*, and *Embelia ribes* using different solvents and four purified compounds (anacardic acid, chloroquinone, glaucarubinone, and methyl gallate) were carried out for their anti-dengue virus (DENV) and anti-chikungunya virus (CHIKV) activities. Maximum nontoxic concentrations of the chloroform, methanol, ethyl acetate, petroleum ether, dichloromethane, and hydroalcoholic extracts of eight plants were used. The antiviral activity was assessed by focus-forming unit assay, quantitative real-time RT-PCR, and immunofluorescence assays. Extracts from *Plumeria alba*, *Ancistrocladus heyneanus*, *Bacopa monnieri*, and *Cucurbita maxima* showed both anti-DENV and CHIKV activity while extract from *Vitex negundo* showed only anti-DENV activity. Among the purified compounds, anacardic acid, chloroquinone and methyl gallate showed anti-dengue activity while only methyl gallate had anti-chikungunya activity. The present study had identified the plant extracts with anti-dengue and anti-chikungunya activities, and these extracts can be further characterized for finding effective phytopharmaceutical drugs against dengue and chikungunya.

## 1 Introduction

Dengue and chikungunya virus infections are important causes of morbidity and mortality in tropical and subtropical parts of the world. Dengue virus (DENV) and chikungunya virus (CHIKV) are transmitted through *Aedes aegypti* and *Aedes albopictus* mosquitoes. Both of the viruses cause acute febrile illness, and symptoms wise, both diseases are identical in the acute phase, though the clinical presentation differs as the infection progresses (Cecilia, 2014).

There are no licensed antivirals/vaccines available against DENV and CHIKV, and their prevention is still based on vector control measures. Therefore, the need for effective drugs with anti-dengue and anti-chikungunya activities is imperative. Natural products from herbal plants have shown to be effective against a variety of viral diseases (Jassim and Naji, 2003). Herbal compounds present an interesting avenue to explore because of their high accessibility in nature and cost effectiveness though safety remains a concern due to lack of sufficient regulatory measures (Ekor, 2014). Moreover, plant extracts are a better source of novel chemical structures with medicinal property which can be exploited to make synthetic drugs with superior activity and reduced toxicity (Choodej et al., 2018). A concerted search involving 3,789 samples from 3,482 plants belonging to 233 families against Ranikhet disease virus, vaccinia virus, Japanese encephalitis virus, and Semiliki forest virus resulted in identifying 242 samples from 96 families with antiviral activity (Dhawan, 2012). A number of plants such as *Rhapis excels, Vernonia amygdalina, Trigonostemon cherrieri, Anacolosa pervilleana*, and *Melia azedarach* L have been reported to possess inhibitory activity against CHIKV (Allard et al., 2012; Bourjot et al., 2012; Sangeetha and Rajarajan, 2015; Chan et al., 2016). Plant-derived compounds such as nobiletin, epigallocatechin-3-gallate, silymarin, curcumin, and harringtonine were reported to have anti-chikungunya activity (Kaur et al., 2013; Lani et al., 2015; Weber et al., 2015; von Rhein et al., 2016; Lin et al., 2017; Mounce et al., 2017). Some other plants like *Cocculus hirsutus*, *Cissampelos pareira*, *Euphorbia hirta*, *Andrographis paniculata*, *Momordica charantia* and *Leucas cephalotes* have been reported to exert antiviral activity against DENV (Tang et al., 2012; Sood et al., 2015; Rajasekaran et al., 2016; Perera et al., 2018; Kaushik et al., 2021; Shukla et al., 2021).

Though a number of plants have been tested for the antiviral activity, it would be worth investigating other plants that have been used in the age-old practices in Ayurveda, which is a traditional form of Indian medicine. In Ayurvedic form of medicine, a group of herbs are combined into formulations called Rasayana which is provided to enhance the body’s resistance to infections and other diseases (Singh et al., 2021). There is a need to study the antiviral activity of those herbs which are used in traditional forms of medicine for which not much scientific evaluations have been carried out. Furthermore, it would be important to evaluate the Indian alternatives to the plants that are not native to India having anti-dengue and anti-chikungunya activities. Therefore, in this study, extracts prepared using seven plants with medicinal properties (**Table 1**) but not tested for their antiviral activity against dengue and chikungunya were investigated for their activity against dengue and chikungunya virus.

**Table 1:** Profile of the herbal plants used in this study.

S. No.	Extract No.	Plant (botanical name)	Family	Local name	Plant part used	Solvent	CC50	Ethnomedicinal use
1	M1C	*Simarouba glauca*	*Simaroubaceae*	Lakshmi taru	Leaves	Chloroform	46.18	Antiparasitic, antipyretic, antidysentric, antihelmenthic, anticancerous, analgesic, antimicrobial (Manasi and Gaikwad, 2011) Antiviral activities (Jose et al., 2020)
2	M1HA	*Simarouba glauca*	*Simaroubaceae*	Lakshmi taru	Leaves	Hydroalcoholic	26.18	
3	M1D	*Simarouba glauca*	*Simaroubaceae*	Lakshmi taru	Leaves	Dichloromethane	31.55	
4	M1E	*Simarouba glauca*	*Simaroubaceae*	Lakshmi taru	Leaves	Ethyl acetate	54.53	
5	M1M	*Simarouba glauca*	*Simaroubaceae*	Lakshmi taru	Leaves	Methanol	48.57	
6	M2C	*Plumeria alba*	*Apocynaceae*	Champa	Bark	Chloroform	66.16	Diarrhea, itching, bronchitis, cough, asthma, bleeding piles, dysentery, tumors, antimicrobial, arthritis (Choudhary et al., 2014a)
7	M2D	*Plumeria alba*	*Apocynaceae*	Champa	Bark	Dichloromethane	90.72	
8	M2E	*Plumeria alba*	*Apocynaceae*	Champa	Bark	Ethyl acetate	90.24	
9	M8D	*Plumeria alba*	*Apocynaceae*	Champa	Leaves	Dichloromethane	63.83	
10	M8E	*Plumeria alba*	*Apocynaceae*	Champa	Leaves	Ethyl acetate	NT	
11	M8M	*Plumeria alba*	*Apocynaceae*	Champa	Leaves	Methanol	NT	
12	M8P	*Plumeria alba*	*Apocynaceae*	Champa	Leaves	Petroleum ether	132.6	
13	M3M	*Anacardium occidentale*	*Anacardiaceae*	Cashew	Leaves	Methanol	196.3	Antibacterial, anti-inflammatory, antihelminthic, antiparastic (Thomas et al., 2015), antiviral (Gonçalves et al., 2005)
14	M3P	*Anacardium occidentale*	*Anacardiaceae*	Cashew	Leaves	Petroleum ether	30.04	
15	M4C	*Vitex negundo*	*Verbenaceae*	Nirgundi	Leaves	Chloroform	35.77	Antimicrobial, anti-inflammatory, anticancer, antiparasitic (Basri et al., 2014), antiviral (Sangeetha and Rajarajan, 2015)
16	M4D	*Vitex negundo*	*Verbenaceae*	Nirgundi	Leaves	Dichloromethane	8.11	
17	M5C	*Ancistrocladus heyneanus*	*Ancistrocladaceae*	Kardal	Bark	Chloroform	16.36	Antimalarial (Bringmann et al., 2013)
18	M5M	*Ancistrocladus heyneanus*	*Ancistrocladaceae*	Kardal	Bark	Methanol	176.2	
19	M6M	*Embelia ribes*	*Primulaceae*	Vidanga	Seeds	Methanol	30.36	Antidiabetic, anticancer, antimicrobial, antifertility (Souravi and Rajasekharan, 2014), antiviral (Hossan et al., 2018)
20	M7M	*Bacopa monnieri*	*Plantaginaceae*	Brahmi	Whole herb	Hydroalcoholic	265.5	Neuroprotective effect, dementia (Saini et al., 2012)
21	M9C	*Cucurbita maxima*	*Cucurbitaceae*	Pumpkin	Seed	Chloroform	49.42	Analgesic and anti-inflammatory (Wahid et al., 2021)
22	M9D	*Cucurbita maxima*	*Cucurbitaceae*	Pumpkin	Seed	Dichloromethane	4.75	
23	M9E	*Cucurbita maxima*	*Cucurbitaceae*	Pumpkin	Seed	Ethyl acetate	10.43	
24	M9M	*Cucurbita maxima*	*Cucurbitaceae*	Pumpkin	Seed	Methanol	NT	
25	M9P	Cucurbita maxima	*Cucurbitaceae*	Pumpkin	Seed	Petroleum ether	741	
26	A-S	Anacardic acid	Sigma				259	Dengue, antihelmintic, antiparasitic (Hundt et al., 2015)
27	C-S	Chloroquinone	Sigma				NT	Dengue, antimalarial (Savarino et al., 2003)
28	MG-S	Methyl gallate	Sigma				66.33	Antiviral (Kane et al., 1988)
29	G-S	Glaucarubinone	Dr. John Beutler’s Lab, NIH USA				663.9	Anticancer, antiparasitic activities (Manasi and Gaikwad, 2011)

NT, not toxic.

## 2 Materials and Methods

### 2.1 Cells, Virus, and Plant Materials

Vero (ATCC No. CCL-81) cell line was maintained using MEM (Himedia, Mumbai, India), supplemented with 10% FBS (Gibco, USA), and antibiotic–antimycotic solution (Sigma-Aldrich, Saint Louis, MO, USA) at 37°C and 5% CO_2_. Dengue virus (DENV) serotype-2 (Strain No. 803347) and chikungunya (CHIKV, Strain No. 061573, P-2, African genotype) were used for this study. DENV-2 stock was prepared in C6/36 (mosquito cell line) and CHIKV was propagated in Vero cells and stored at −80°C.

A total of 25 different parts from eight plants, i.e., *Vitex negundo* (RMRC-1355), *Plumeria alba* (RMRC-1357), *Ancistrocladus heyneanus* (RMRC-1359), *Bacopa monnieri *(RMRC-1361), *Anacardium occidentale* (RMRC-1356), *Cucurbita maxima* (RMRC-1362), *Simarouba glauca* (RMRC-1358), and *Embelia ribes* (RMRC-1360) (**Table 1**), selected based on their ethno botanical use in the treatment of infectious diseases, were collected from various sites in Belagavi, identified, and authenticated at ICMR-National Institute of Traditional Medicine, Belagavi, where voucher herbarium specimens were deposited. Four purified compounds, i.e., anacardic acid (Sigma-Aldrich, Saint Louis, MO, USA), chloroquinone (Sigma-Aldrich, Saint Louis, MO, USA), methyl gallate (Sigma-Aldrich, Saint Louis, MO, USA), and glaucarubinone (Dr. John Beutler**’**s Lab, NIH USA) were also included in this study.

### 2.2 Preparation of Plant Extracts

Plant parts (leaves, bark, seeds, and whole herbs) were air-dried, ground to powder, and macerated in different solvents such as ethanol (99.9% pure; Changshu Hongsheng Fine Chemical Co. Ltd., Jiangshu Province), methanol (99.5% pure; Fisher Scientific, Mumbai, India), petroleum ether (99.9% pure; Fisher Scientific, Mumbai, India), ethyl acetate (99.0% pure; Fisher Scientific, Mumbai, India), chloroform (99.7% pure; Fisher Scientific, Mumbai, India), and dichloromethane (99.5% pure; Fisher Scientific, Mumbai, India). Each solvent extract was extracted three times after 72 h of maceration. The extract was filtered through Whatman filter paper No. 1, and the solvent was evaporated using a rotary evaporator at different temperatures (as per the solvent property) and further extracts were stored at −4°C.

### 2.3 Herbal Extracts Stock Solution

A total of 25 extracts and four purified compounds were used for further screening (**Table 1**). Extracts and stock solutions of purified compounds (1 mg/ml) were prepared by diluting extracts and compounds in different solvents, i.e., petroleum ether, methanol, ethanol, and dimethyl sulfoxide (0.1%) and purified by filtering through a syringe filter (pore size = 0.2 µM), and the solutions were preserved at −20°C until use.

### 2.4 Cell Cytotoxicity Assays

The cytotoxicity effect of the extracts and the compounds was evaluated by 3-(4,5-dimethythiazol-2-yl)-2,5-diphenyl tetrazolium bromide (MTT) assay as described previously (Parashar et al., 2013). Briefly, monolayers of Vero cells in 96-well plates were incubated with different concentrations (0 to 200 µg/ml) of test formulations for 5 days at 37°C, incubated with MTT solution (5 mg/ml) for an additional 3 h at 37°C. The solubilized formazan crystals were measured using a microplate reader (BioTek Synergy, USA) at 570 nm. The percentage inhibition and CC50 values were calculated.

### 2.5 Primary Screening and Antiviral Assay Against DENV-2 and CHIKV

All the extracts at their maximum nontoxic dose were assayed for their antiviral activity against DENV and CHIKV under posttreatment condition as described earlier (Panda et al., 2021; Patil et al., 2021).

The extracts which showed antiviral activity were further tested at different concentrations for their antiviral activity under all the three treatment conditions (pre-, co-, and posttreatment) as described earlier (Panda et al., 2021; Patil et al., 2021).

During pretreatment, the cells were pretreated with a formulated extracts at 37°C for 24 h followed by removal of the culture supernatant, the cells were then infected either with DENV-2 or CHIKV and incubated for 1 h at 37°C. Any unbound virus particles were removed by PBS, washed two times, and incubated after the addition of maintenance media (MEM with antibiotics and 2% FBS).

In cotreatment, the virus was mixed with different concentrations of the formulated extracts and the mixture was used for infecting cells for a duration of 1 h.

For posttreatment, the cells were infected with DENV-2 or CHIKV for 1 h and treated with the formulated extracts after 24 h.

For all treatments, the plates were incubated after infection for 120 h in the case of DENV-2 and 48 h for CHIKV. After incubation, the plates were frozen at −80°C and thawed to collect culture supernatant for estimation of virus titer by focus-forming unit (FFU) assay. All the experiments were performed in triplicates. For significant results, the experiments were repeated again in triplicates.

Irrespective of the type of treatment, 0.1 multiplicity of infection (MOI) of DENV-2 or 0.01 MOI of CHIKV was used for infection. MOI was calculated on the basis of number of cells used for seeding the wells.

The tissue culture supernatants collected from the different wells treated under different conditions were assessed for viral genomic RNA using quantitative real-time RT-PCR and infectious virus particle titer using FFU assay. The percent of cells infected was assessed using immunofluorescence assay (IFA).

### 2.6 Quantitative Real-Time RT-PCR, FFU assay, and Immunofluorescence Assay

Detection and quantitative estimation of viral genomic RNA was done using quantitative real-time RT-PCR assay. The primers and probes used for amplifying DENV-2 and CHIKV and PCR conditions have been described earlier (Panda et al., 2021; Patil et al., 2021). The viral RNA load of the samples were calculated based on a standard graph generated using cycle threshold values of tenfold dilutions of *in vitro* transcribed viral RNA with known copy numbers. IFA and FFU assays for DENV-2 and CHIKV were performed as described earlier.

### 2.7 Statistical Analysis

The virus output was measured in terms of FFU/ml or viral RNA copies/ml. The test conditions were compared with virus control using one-way ANOVA followed by multiple comparisons. A *p*-value of less than 0.05 was considered significant. All analyses were performed using GraphPad Prism software version 7.

## 3 Results

### 3.1 Profile of Medicinal Plants Used in the Study

In this study, chloroform, methanol, ethyl acetate, petroleum ether, dichloromethane, and hydroalcoholic extracts of eight plants (*Vitex negundo*, *Plumeria alba*, *Ancistrocladus heyneanus*, *Bacopa monnieri*, *Anacardium occidentale*, *Cucurbita maxima*, *Simarouba glauca*, and *Embelia ribes*) were tested for their antiviral activity against DENV and CHIKV. The profile of the medicinal plants used in this study is listed in **Table 1**.

### 3.2 Evaluation of the Cytotoxicity of Plant Extracts

Potential cytotoxic effects of the 25 extracts and four purified compounds were determined using the MTT assay in Vero CCL-81 cells. Three extracts (M8E, M8M, and M9M) and one purified compound (G-S) showed no cytotoxicity (**Table 1**). The CC50 values of the different extracts are given in **Table 1**. The concentration of the extract which allowed around 80% cell viability was further used for studying the antiviral activity. The cutoff of 80% viability was decided as per earlier literature (ISO 10993-5:2009; Wang et al., 2018). The effect of different extracts at different concentrations on cell viability with CC50 values is provided in **Supplementary Figure S1**.

### 3.3 Primary Antiviral Screening of Compounds Against DENV and CHIKV

**Figure 1:**
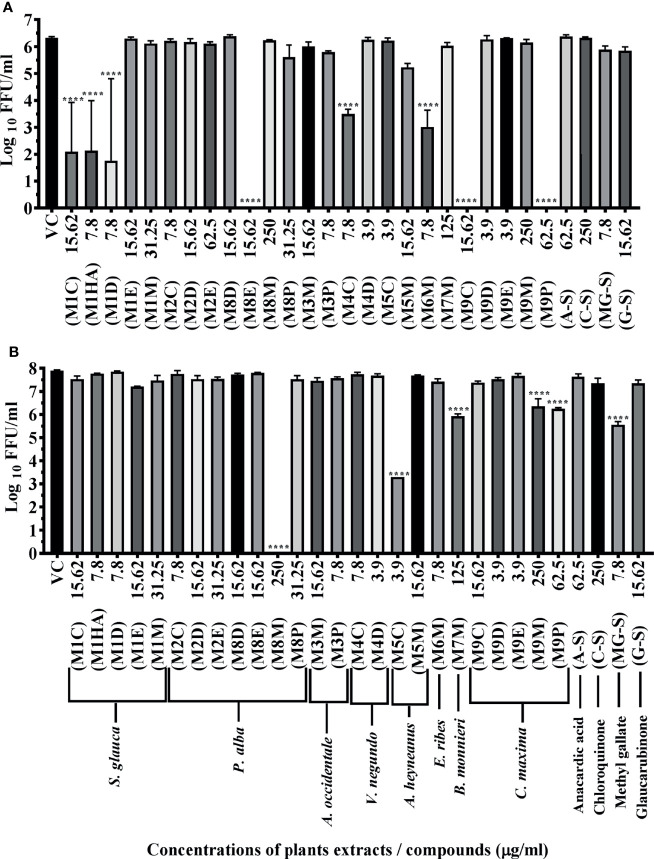
Antiviral screening of different plant extracts at maximal nontoxic concentration against DENV **(A)** and CHIKV **(B)** under posttreatment condition. Vero CCL-81 cells were treated with highest maximum nontoxic dose of extracts 24 h postinfection and incubated for 96 h for DENV **(1A)** and 24 h in the case of CHIKV **(1B)**, and after the incubation, the plates were frozen and the culture filtrates were used for the different assays. The experiments were performed at two independent time points in triplicates, and the results are expressed as mean log_10_ focus-forming unit/ml ± standard error. All the treatment conditions were compared with the virus control. ^****^ *p* < 0.0001.

The plant extracts were screened by assessing their antiviral activity against DENV and CHIKV postinfection. FFU assay was used to measure the titer of the virus. The highest nontoxic dose (which allowed around 80% cell viability) was used for all extracts. Out of 25 extracts and four purified compounds, five extracts (M2C, M8M, M4C, M5C, and M7M) and three purified compounds [anacardic acid (A-S), chloroquinone (C-S), and methyl gallate (MG-S)] showed significant reduction (≥1 log_10_ reduction) in the titer of DENV compared with virus control (infected cells without any treatment) (**Figure 1A**). Four extracts (M8M, M5C, M7M, and M9M) and one purified compound (MG-S) affected CHIKV titer (**Figure 1B** and **Table 2**).

**Table 2:** Summary of effective extracts showing inhibition of DENV and CHIKV under different treatment conditions.

S. No.	Extracts name	Plant (botanical name)	Solvent used for extraction	Maximum concentration (µg/ml)	Log difference effectiveness against DENV	Log difference effectiveness against CHIKV
1	M2C	*Plumeria alba*	Chloroform	7.8	Cotreatment: 1.022 Posttreatment: 5.108	–
2	M8M	*Plumeria alba*	Methanol	250	Cotreatment: 2.096 Posttreatment: 1.285	Pretreatment: 7.87 Posttreatment: 7.564
3	M4C	*Vitex negundo*	Chloroform	7.8	Cotreatment: 2.432 Posttreatment: 2.224	–
4	M5C	*Ancistrocladus heyneanus*	Chloroform	3.9	Posttreatment: 5.108	Pretreatment: 2.536 Posttreatment: 3.327
5	M7M	*Bacopa monnieri*	Hydroalcoholic	125	Cotreatment: 2.187 Posttreatment: 5.108	Posttreatment: 1.311
6	M9M	*Cucurbita maxima*	Methanol	250	–	Pretreatment: 7.878 Cotreatment: 7.569 Posttreatment: 7.564
7	A-S	–	–	62.5	Posttreatment: 5.108	–
8	C-S	–	–	250	Pretreatment: 1.912 Cotreatment: 1.062 Posttreatment: 5.108	–
9	MG-S	–	–	7.8	Pretreatment: 1.006 Posttreatment: 5.108	Posttreatment: 2.16

### 3.4 Effect of Plant Extracts on DENV and CHIKV Under Different Conditions and Concentrations

#### 3.4.1 *Plumeria alba* (M2C and M8M)

**Figure 2:**
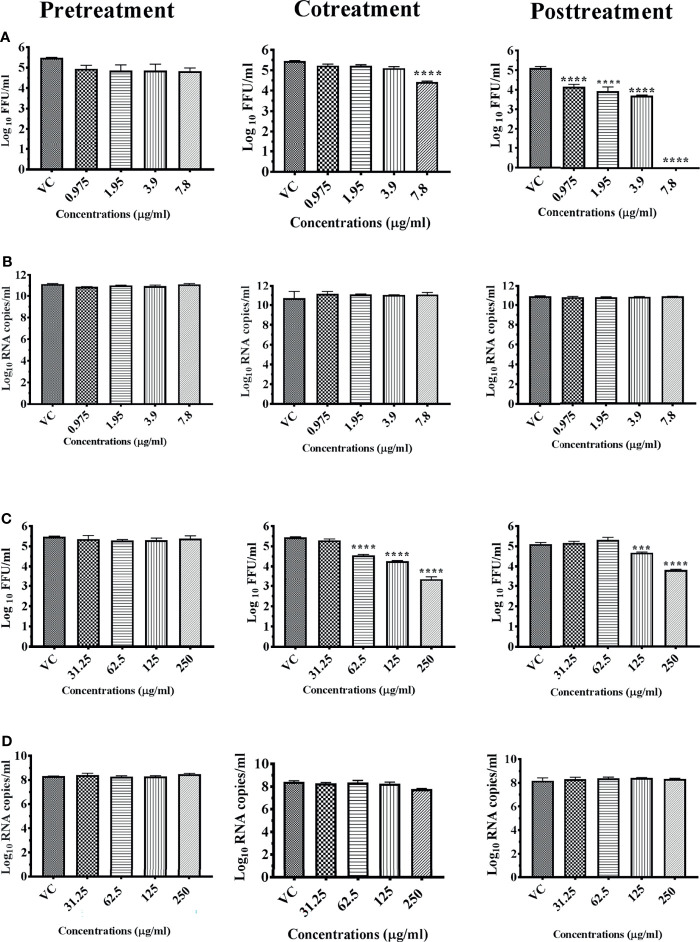
Antiviral effect of *Plumeria alba* bark extract prepared using chloroform (M2C) and leaf-based extract prepared using methanol (M8M) against DENV under different treatment conditions. Vero CCL-81 cells were pre-, co-, and posttreated with different concentrations of extracts, and 120 h incubation after infection, the plates were frozen and the culture filtrates were used for the FFU assay (**A**, M2C; **C**, M8M) and real-time PCR (**B**, M2C; **D**, M8M). The experiments were performed at two independent time points in triplicates, and the results are expressed as either mean log_10_ focus-forming unit/ml ± standard error (**A**, M2C; **C**, M8M) or mean log_10_ viral RNA copies/ml ± standard error (**B**, M2C; **D**, M8M). All the treatment conditions were compared with the virus control. ^****^ *p* < 0.0001; ^***^ *p* < 0.001.

Chloroform extract of *Plumeria alba* bark (M2C) and methanol extract of *Plumeria alba* (M8M) were processed to check their prophylactic (pretreatment effect), virucidal (cotreatment effect), and therapeutic (posttreatment effect) activities at concentrations of ≤7.8 and ≤250 μg, respectively, and the virus titer was determined by FFU assay and viral RNA by quantitative real-time RT-PCR. Both M2C and M8M did not show any anti-DENV activity under pretreatment condition (**Figures 2A, C** **)**. Under cotreatment condition, M2C exerted significant one log (5.451 to 4.429 mean log_10_ FFU/ml) reduction at a concentration of 7.8 μg while M8M showed a significant dose-dependent reduction from a concentration of 62.5 μg onwards with two log reductions (5.451 to 3.355 mean log_10_ FFU/ml) at 250 μg concentration in DENV titer compared with virus control (**Figures 2A, C** **)**. Under posttreatment condition, M2C showed a dose-dependent reduction from 0.975 μg concentration onwards with 100% reduction at a concentration of 7.8 μg in DENV titer compared with virus control (*p* < 0.0001) (**Figure 2A**). In comparison with virus control, M8M exerted a dose-dependent reduction from 125 μg onwards with more than one log reduction (5.108 to 3.822 mean log_10_ FFU/ml) at a concentration of 250 μg (**Figure 2C**). Both the extracts did not show any reductions in the viral RNA titer as assessed by quantitative real-time RT-PCR under all conditions (**Figures 2B, D** **)**.

**Figure 3:**
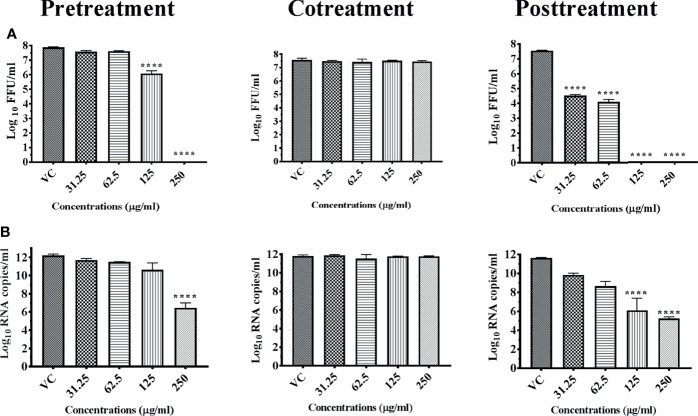
Antiviral effect of bark and leaves of *Plumeria alba* methanol extract (M8M) against CHIKV under different treatment conditions. Vero CCL-81 cells were pre-, co-, and posttreated with different concentrations of extracts, and 48 h incubation after infection, the plates were frozen and the culture filtrates were used for the FFU assay **(A)** and real-time PCR **(B)**. The experiments were performed at two independent time points in triplicates, and the results are expressed as either mean log_10_ focus-forming unit/ml ± standard error **(A)** or mean log_10_ viral RNA copies/ml ± standard error **(B)**. All the treatment conditions were compared with the virus control. ^****^ *p* < 0.0001.

In the case of chikungunya, 250 μg methanol extract of *Plumeria alba* leaves (M8M) showed 100% reduction of CHIKV titer under pre- and posttreatment conditions compared with virus control (*p* < 0.0001). The effect was more pronounced under posttreatment condition, and a significant dose-dependent reduction was observed from with 31.25 μg onwards compared with virus control (*p* < 0.0001) (**Figure 3A**). Quantitative real-time RT-PCR results revealed a significant reduction in viral RNA titer under both pre- and posttreatment conditions at concentration of 250 and 125 μg, respectively, compared with the untreated (**Figure 3B**) ones. A lack of anti-chikungunya activity was observed under cotreatment condition at the level of viral RNA and focus-forming units (*p* > 0.05) (**Figures 3A, B** **)**.

#### 3.4.2 *Vitex negundo* (M4C)

**Figure 4:**
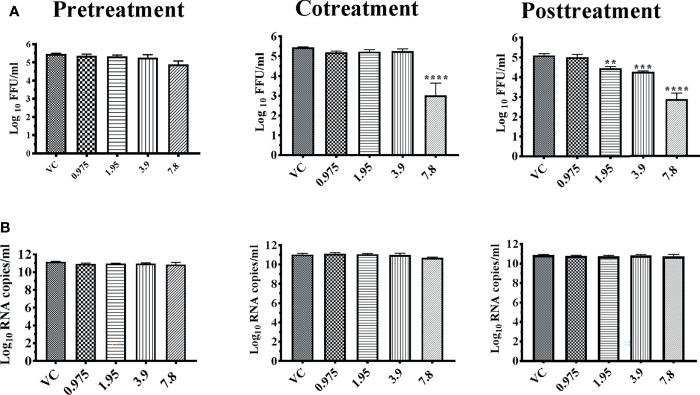
Antiviral effect of leaves of *Vitex negundo* chloroform extract (M4C) against DENV under different treatment conditions. Vero CCL-81 cells were pre-, co-, and posttreated with different concentrations of extracts, and 120 h incubation after infection, the plates were frozen and the culture filtrates were used for the FFU assay **(A)** and real-time PCR **(B)**. The experiments were performed at two independent time points in triplicates, and the results are expressed as mean log_10_ focus-forming unit/ml ± standard error as well as mean log_10_ viral RNA copies/ml ± standard error. All the treatment conditions were compared with the virus control. ^****^ *p* < 0.0001; ^***^ *p* < 0.001; ^**^ *p* < 0.005.

The chloroform extract of *Vitex negundo* leaves showed more than two log reductions of DENV titer in case of cotreatment (5.451 to 3.019 mean log_10_ FFU/ml) and posttreatment (5.108 to 2.883 mean log_10_ FFU/ml) compared with respective virus controls at 7.8 μg concentration (**Figure 4A**). The antiviral effect was more pronounced under posttreatment condition and a dose-dependent inhibitory effect on virus was observed from 1.958 μg concentration onwards. Quantitative real-time RT-PCR results revealed that the extract had no effect on viral RNA titer under all conditions (**Figure 4B**), and a significant reduction was observed for viral RNA using quantitative real-time RT-PCR (**Figure 4B**). The extract lacked anti-CHIKV activity.

#### 3.4.3 *Ancitrocladus heyeanus* (M5C)

**Figure 5:**
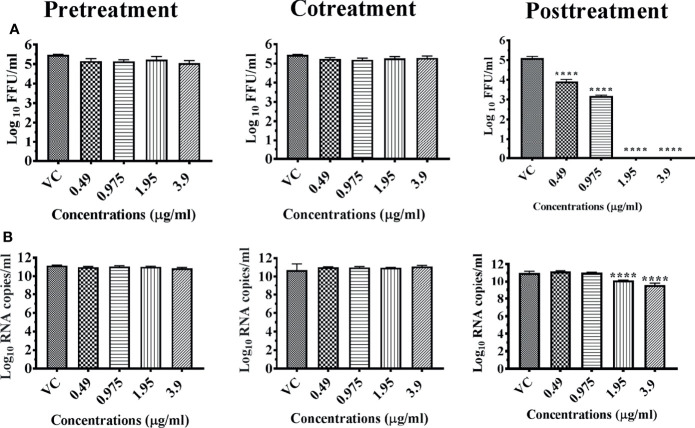
Antiviral effect of bark of *Ancitrocladus heyneanus* chloroform extract (M5C) against DENV under different treatment conditions. Vero CCL-81 cells were pre-, co-, and posttreated with different concentrations of extracts, and 120 h incubation after infection, the plates were frozen and the culture filtrates were used for the FFU assay **(A)** and real-time PCR **(B)**. The experiments were performed at two independent time points in triplicates, and the results are expressed as either mean log_10_ focus-forming unit/ml ± standard error **(A)** or mean log_10_ viral RNA copies/ml ± standard error **(B)**. All the treatment conditions were compared with the virus control. ^****^ *p* < 0.0001.

Anti-dengue and anti-chikungunya activities of chloroform extract of *Ancitrocladus heyeanus* bark was investigated under pre-, co-, and posttreatment conditions at concentrations of ≤3.9 μg which is the maximum nontoxic concentration. The results revealed that the extract had a dose-dependent anti-DENV activity from 0.49 μg onwards under posttreatment condition. Complete reduction in viral titer was observed at 1.95 and 3.9 μg concentrations (**Figure 5A**). Though not prominent as infectious virus particles, a reduction in viral RNA titer was observed from 1.95 μg onwards (**Figure 5B**).

**Figure 6:**
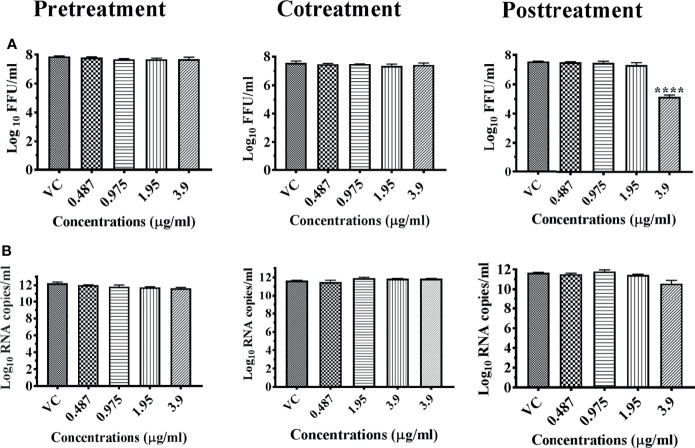
Antiviral effect of bark of *Ancitrocladus heyneanus* chloroform extract (M5C) against CHIKV under different treatment conditions. Vero CCL-81 cells were pre-, co-, and posttreated with different concentrations of extracts, and 48 h incubation after infection, the plates were frozen and the culture filtrates were used for the FFU assay **(A)** and real-time PCR **(B)**. The experiments were performed at two independent time points in triplicates, and the results are expressed as either mean log_10_ focus-forming unit/ml ± standard error **(A)** or mean log_10_ viral RNA copies/ml ± standard error **(B)**. All the treatment conditions were compared with the virus control. ^****^ *p* < 0.0001.

For chikungunya, M5C at a concentration of 3.9 μg showed a reduction in viral titer from 7.563 to 2.430 mean log_10_ FFU/ml under posttreatment condition. However, similar reduction in viral RNA titer was not observed. The extract lacked anti-chikungunya activity under pre- and cotreatment conditions (**Figures 6A, B** **)**.

#### 3.4.4 Bacopa monnieri (M7M)

**Figure 7:**
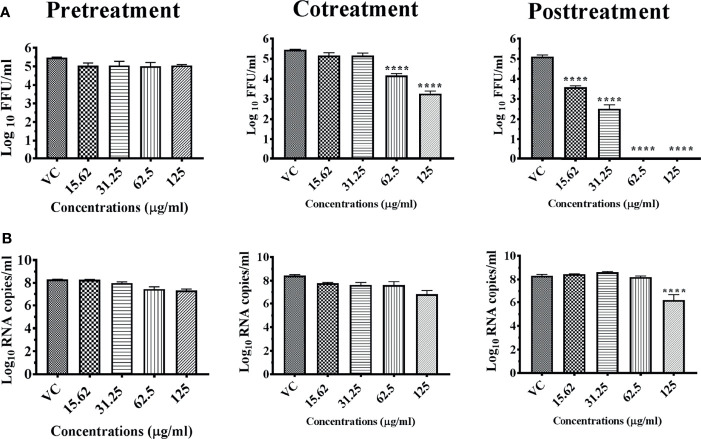
Antiviral effect of whole herb of *Bacopa monnieri* hydroalcoholic extract (M7M) against DENV under different treatment conditions. Vero CCL-81 cells were pre-, co-, and posttreated with different concentrations of extracts, and 120 h incubation after infection, the plates were frozen and the culture filtrates were used for the FFU assay **(A)** and real-time PCR **(B)**. The experiments were performed at two independent time points in triplicates, and the results are expressed as either mean log_10_ focus-forming unit/ml ± standard error **(A)** or mean log_10_ viral RNA copies/ml ± standard error **(B)**. All the treatment conditions were compared with the virus control. ^****^ *p* < 0.0001.

Under cotreatment condition, hydroalcoholic extract of *Bacopa monnieri* whole herb (M7M) reduced the DENV titer starting from 62.5 μg onwards with a maximum reduction (5.451 to 3.264 mean log_10_ FFU/ml) at 125 μg under cotreatment condition. The anti-dengue activity was more pronounced under posttreatment condition with a reduction starting from 15.62 μg, and a complete reduction of DENV titer was observed from 62.5 μg onwards (**Figure 7A**). However, the corresponding reduction in viral RNA titer was not observed for both co- and posttreatment conditions (**Figure 7B**).

**Figure 8:**
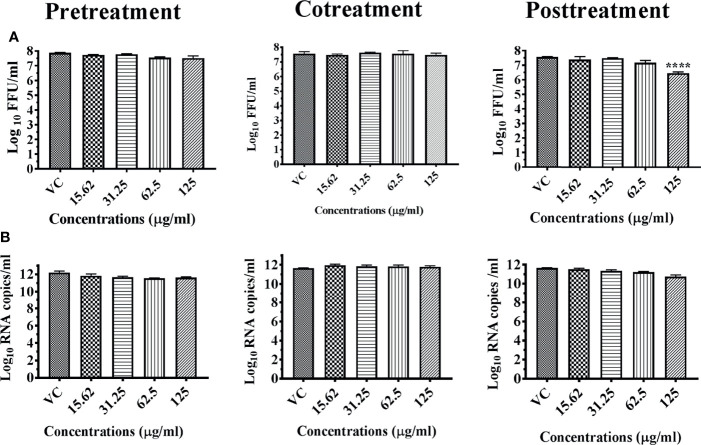
Antiviral effect of whole herb of *Bacopa monnieri* hydroalcoholic extract (M7M) against CHIKV under different treatment conditions. Vero CCL-81 cells were pre-, co-, and posttreated with different concentrations of extracts, and 48 h incubation after infection, the plates were frozen and the culture filtrates were used for the FFU assay **(A)** and real-time PCR **(B)**. The experiments were performed at two independent time points in triplicates, and the results are expressed as mean log_10_ focus-forming unit/ml ± standard error as well mean log_10_ viral RNA copies/ml ± standard error. All the treatment conditions were compared with the virus control. ^****^ *p* < 0.0001.

In the case of chikungunya, more than one log reduction (7.563 to 6.451 mean log_10_ FFU/ml) under posttreatment condition was observed at the maximum nontoxic concentration of 125 μg (**Figure 8A**). No significant reduction of viral RNA was observed for viral RNA under all conditions (**Figure 8B**).

#### 3.4.5 Cucurbita maxima (M9M)

**Figure 9:**
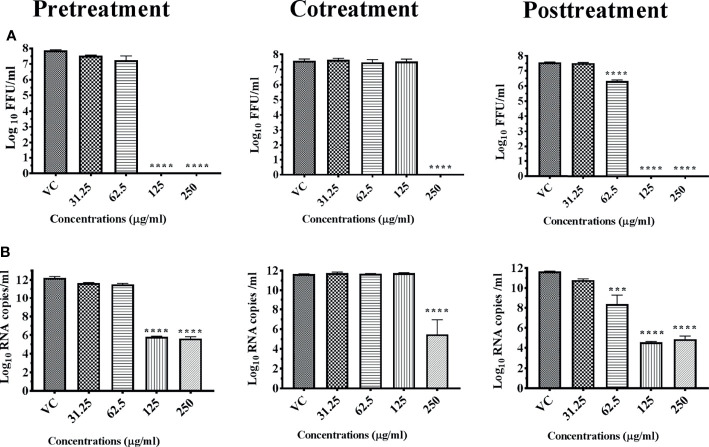
Antiviral effect of *Cucurbita maxima* seed methanol extract (M9M) against CHIKV under different treatment conditions. Vero CCL-81 cells were pre-, co-, and posttreated with different concentrations of extracts, and 48 h incubation after infection, the plates were frozen and the culture filtrates were used for the FFU assay **(A)** and real-time PCR **(B)**. The experiments were performed at two independent time points in triplicates, and the results are expressed as either mean log_10_ focus-forming unit/ml ± standard error **(A)** or mean log_10_ viral RNA copies/ml ± standard error **(B)**. All the treatment conditions were compared with the virus control. ^****^ *p* < 0.0001; ^***^ *p* < 0.001.

Methanol extract of *Cucurbita maxima* seeds (M9M) exerted anti-chikungunya activity under all conditions. The antiviral effect was observed under pre- and posttreatment conditions at concentrations above 125 µg with complete reduction in viral titer (**Figure 9A**). Under cotreatment condition, complete reduction in viral titer was observed at 250 µg concentration. Corresponding reduction in CHIKV RNA titer was also observed under all conditions though not as the same extent to that infectious virus titer (**Figure 9B**).

### 3.5 Effect of Pure Compounds on DENV and CHIKV Under Different Conditions and Concentrations

#### 3.5.1 Anacardic Acid

**Figure 10:**
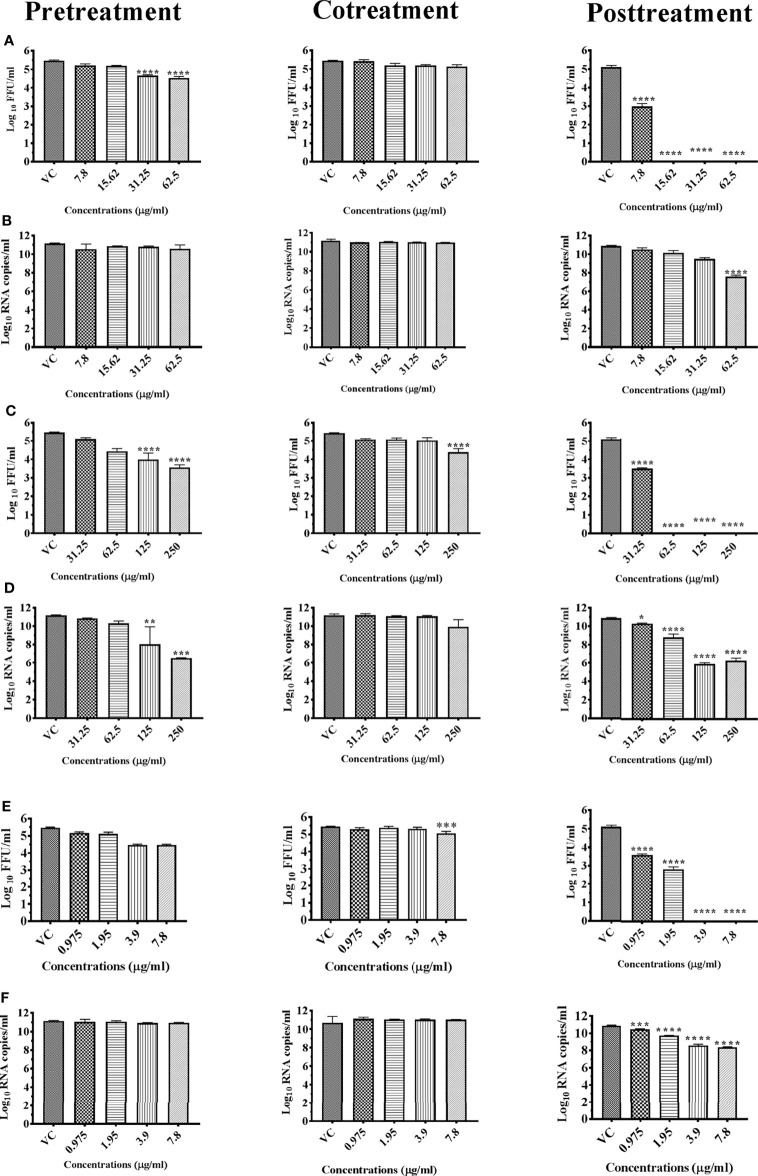
Antiviral effect of pure compounds anacardic acid (A-S), chloroquinone (C-S), and methyl gallate (MG-S) against DENV under different treatment conditions. Vero CCL-81 cells were pre-, co-, and posttreated with different concentrations of pure compounds, and 120 h incubation after infection, the plates were frozen and the culture filtrates were used for the FFU assay [**(A)**, A-S; **(C)**, C-S; and **(E)**, MG-S] and real-time PCR [**(B)**, for A-S; **(D)**, C-S; **(F)**, MG-S]. The experiments were performed at two independent time points in triplicates, and the results are expressed as either mean log_10_ focus-forming unit/ml ± standard error [**(A)**, A-S; **(C)**, C-S; and **(E)**, MG-S] or mean log_10_ viral RNA copies/ml ± standard error [**(B)**, A-S; **(D)**, C-S; and **(F)**, MG-S]. All the treatment conditions were compared with the virus control. ^****^ *p* < 0.0001; ^***^ *p* < 0.001; ^**^ *p* < 0.005; ^*^ *p* < 0.05.

Among the pure compounds, A-S exerted anti-dengue activity under pre- and posttreatment conditions. The effect was prominent under posttreatment condition in which the anti-dengue activity was observed from 7.8 µg onwards with complete reduction in infectious virus titer from 15.62 µg onwards. Under pretreatment condition, the mild reduction in virus titer was observed at concentrations of 31.25 and 62.5 µg (**Figure 10A**). A mild but significant reduction in DENV RNA titer was observed under posttreatment condition at 62.5 µg (*p* < 0.0001) (**Figure 10B**). Anacardic acid did not exert anti-chikungunya effect.

#### 3.5.2 Chloroquinone

C-S exerted anti-dengue activity under all three conditions. Under pretreatment condition, the activity was observed from 62.5 µg onwards while under cotreatment condition, the activity was observed at 250 µg. The anti-dengue activity was more prominent under posttreatment condition in which anti-dengue activity was observed from 31.25 µg onwards with complete inhibition of virus titer from 61.25 µg onwards (**Figure 10C**). A significant decrease in DENV RNA titer was also observed under pre- and posttreatment conditions though not as prominent as the decrease in infectious virus titer was observed (**Figure 10D**). Chloroquinone did not show any anti-chikungunya activity.

#### 3.5.3 Methyl Gallate

MG-S decreased DENV titer in a dose-dependent manner from 0.975 µg onwards and a 100% reduction in FFU was observed compared with virus control at concentrations of 3.9 and 7.8 µg under posttreatment condition. Under pretreatment and cotreatment conditions, a mild reduction in FFU titer was observed at 7.8 µg (**Figure 10E**).

**Figure 11:**
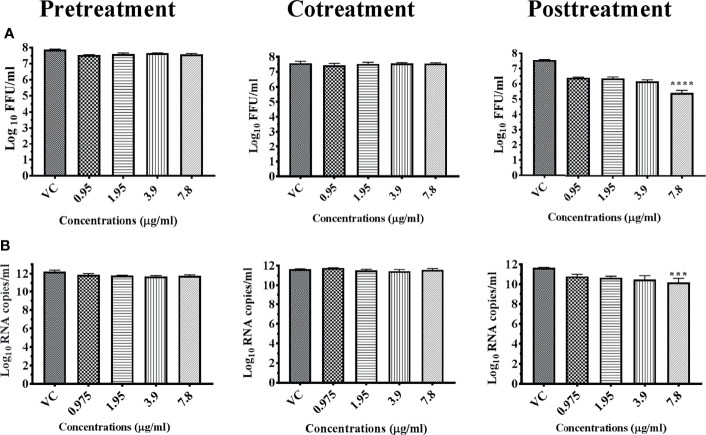
Antiviral effect of pure compound methyl gallate (MG-S) against CHIKV under different treatment conditions. Vero CCL-81 cells were pre-, co-, and posttreated with different concentrations of pure extract, and 48 h incubation after infection, the plates were frozen and the culture filtrates were used for the FFU assay **(A)** and real-time PCR **(B)**. The experiments were performed at two independent time points in triplicates, and the results are expressed as either mean log_10_ focus-forming unit/ml ± standard error **(A)** or mean log_10_ viral RNA copies/ml ± standard error **(B)**. All the treatment conditions were compared with the virus control. ^****^ *p* < 0.0001; ^***^ *p* < 0.001.

In the case of chikungunya, methyl gallate exerted a mild reduction virus titer in terms of FFU and viral RNA at a concentration of 7.8 µg under posttreatment conditions (**Figures 11A, B** ).

### 3.6 Effect of Plant Extracts and Compounds on DENV and CHIKV Infection in Vero Cells Under Posttreatment Condition Using IFA

**Figure 12:**
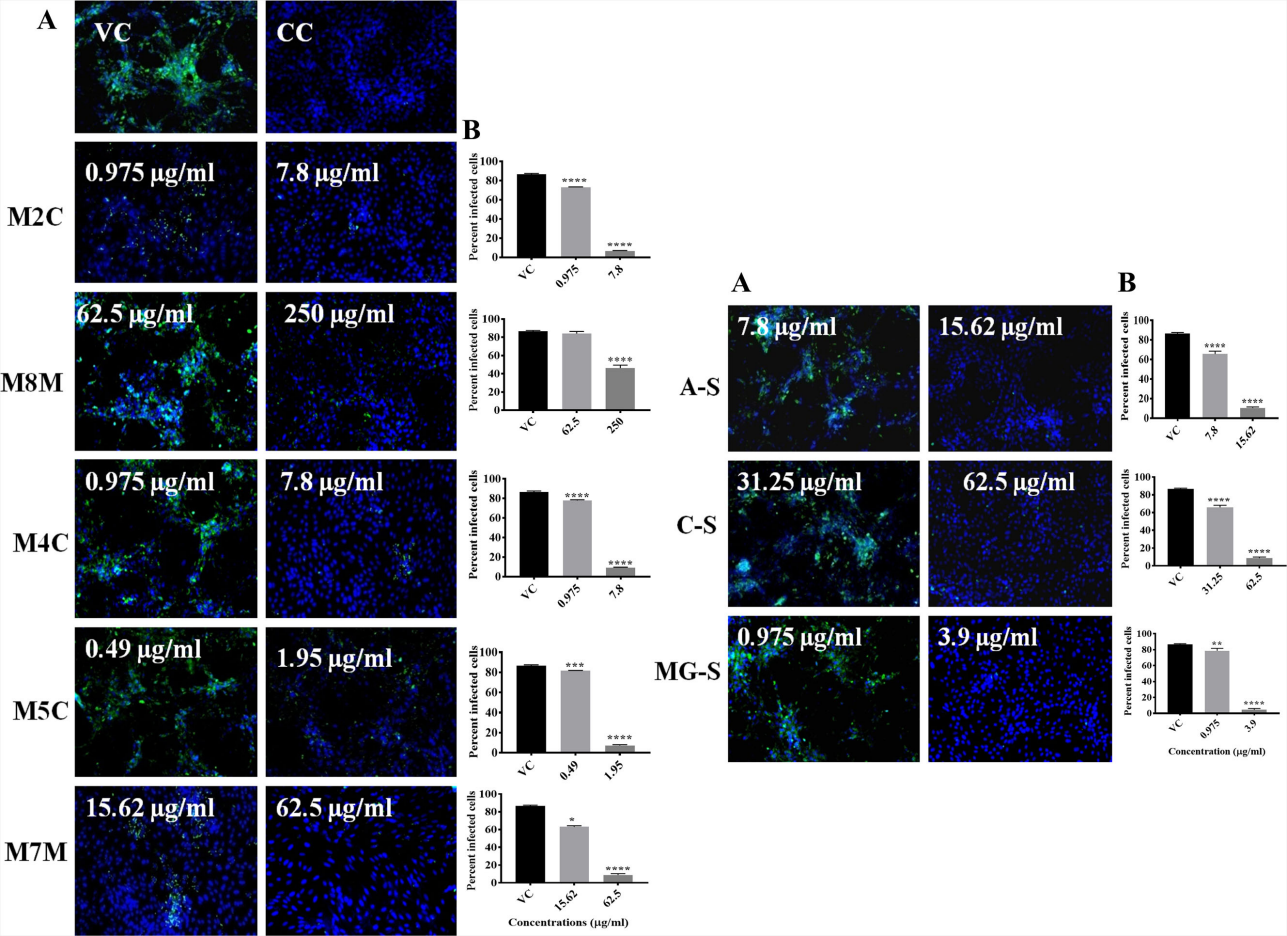
Immunofluorescence assay images for DENV after treatment with extracts and compounds. Images represent DENV-2-infected Vero CCL-81 cell lines under posttreatment condition. Virus-infected cells appear green in color **(A)**. Percentage of infected Vero CCL-81 cell line in cultures infected with virus with different concentrations of extracts under posttreatment condition **(B)**. All the treatment conditions were compared with the virus control. ^****^ *p* < 0.0001; ^***^ *p* < 0.001; ^**^ *p* < 0.005; ^*^ *p* < 0.05. VC, virus control; CC, cell control.

**Figure 13:**
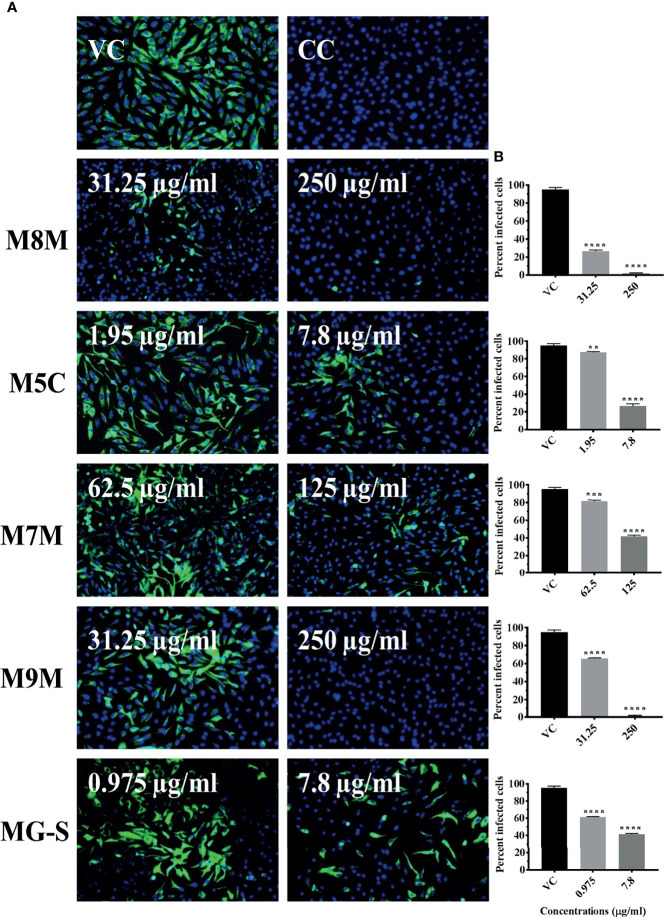
Immunofluorescence assay images for CHIKV after treatment with extracts and compounds. Images represent CHIKV-infected Vero CCL-81 cell lines under posttreatment condition. Virus-infected cells appear green in color **(A)**. Percentage of infected Vero CCL-81 cell line in cultures infected with virus with different concentrations of extracts under posttreatment condition **(B)**. All the treatment conditions were compared with the virus control. ^****^ *p* < 0.0001; ^***^ *p* < 0.001; ^**^ *p* < 0.005. VC, virus control; CC, cell control.

The anti-dengue activity exerted by the extracts and compounds postinfection was further confirmed by IFA. IFA results revealed that the extracts and compounds significantly reduced the percent of infected cells compared with virus control (**Figure 12**). IFA results confirmed the anti-chikungunya activity of M8M, M5C, M7M, M9M, and MG-S which was evident by the low percent of infection in wells which were subjected to treatment with extracts and compounds postinfection (**Figure 13**).

## 4 Discussion

Dengue and chikungunya diseases are serious public health problems, and the unavailability of antivirals make these diseases a concern. Traditionally, plants have been used to treat various diseases including viral diseases for centuries (Herrmann et al., 2011; Ogbole et al., 2018). Therefore, plant-based anti-chikungunya and anti-dengue drugs might be an alternative option to treat these mosquito-borne diseases.

In the present study, chloroform, methanol, ethyl acetate, hydro-alcoholic, petroleum ether, and dichloromethane extracts of five selected plant species (*Plumeria alba*, *Ancistrocladus heyneanus*, *Bacopa monnieri*, *Curcubita maxima*, *Vitex negundo*) were tested for their antiviral activity against DENV and CHIKV. Out of 25 extracts and four purified compounds, five extracts (M2C, M8M, M4C, M5C, and M7M) and three purified compounds (A-S, C-S, and MG-S) showed anti-dengue activity, while four extracts (M8M, M5C, M7M, and M9M) and one purified compound (MG-S) exerted significant anti-chikungunya activity.

The chloroform extract from *Plumeria alba* bark (M2C) showed 100% activity against DENV under posttreatment conditions and methanol extract from *Plumeria alba* leaves (M8M) showed 100% activity against CHIKV. The results suggest the therapeutic utility of *Plumeria alba* against both of these viruses. Since hydroalcoholic extracts of *Plumeria alba* leaves have been reported to possess antiarthritic activity, it might also be useful in reducing arthritis in CHIKV-infected patients (Choudhary et al., 2014b). Earlier, the inhibitory activity of *Plumeria rubra* containing fulvoplumierin against human immunodeficiency virus type 1 (HIV) reverse transcriptase has been reported (Tan et al., 1991). Plumericin compounds isolated from *Plumeria* species have been reported to inhibit *Leishmania donovani* and *Candida* species (Sharma et al., 2011; Singh et al., 2011). *Plumeria alba* is known to have various chemical substances including compounds with sterol-like structures like taraxerol, lupeol, betuline, coumarone, and fulvoplumericin which could contribute to antiviral activity and need further investigations (Anggoro et al., 2020).

Whole herb of *Bacopa monnieri* (M7M) also showed a significant reduction of DENV and CHIKV titers under posttreatment conditions. *Bacopa monnieri*, commonly known as Brahmi, is commonly used in the Indian traditional system of medicine as a memory enhancer (Shinomol et al., 2012). It also has been reported to possess anti-inflammatory, analgesic, antipyretic, sedative, and antiepileptic properties (Saini et al., 2012). *Bacopa monnieri* has been reported to contain bacosides and cucurbitacins which have medicinal properties and needs to be investigated for antiviral activity (Bhandari et al., 2007; Banerjee et al., 2021). Extract from *Ancitrocladus heyneanus* bark (M5C) showed total reduction of viral foci of DENV and significant activity against CHIKV. Plant from *Ancistrocladus* genus has been shown to have anticancer, anti-HIV, antimalarial, and antibacterial activities (Karn et al., 2014). *Ancitrocladus heyaneanus* is reported to have ancisheynine 1 and betulinic acid among which betulinic acid possesses anti-dengue activity (Bringmann et al., 1996; Bringmann et al., 1997; Loe et al., 2020).

Leaves of *Vitex negundo* (M4C) showed a significant reduction of DENV, while no significant activity was found against CHIKV. The ethanolic extract of *Vitex negundo* has been reported to inhibit the Asian genotype strain of CHIKV (Kothandan and Swaminathan, 2014). However, in the present study, ECSA genotype of CHIKV was used. A flavone named vitexicarpone, ursolic acid, and betulinic acid have been isolated from *Vitex negundo* leaves, and the presence of betulinic acid might explain its anti-dengue activity (Chandramu et al., 2003, Díaz et al., 2003).

Seeds of *Curcubita maxima* (M9M) showed a 100% reduction in the case of CHIKV. *Cucurbita* leaves contain iron and vitamins (Orech et al., 2007; Nwaoguikpe et al., 2013) and have been reported to possess the potential to treat anemia and sickle cell anemia (Manokaran et al., 2010; Nwaoguikpe et al., 2013). *Curcubita maxima* seeds are also known to exert anti-platelet activity (Sanzana et al., 2021).

The purified compounds *viz.* anacardic acid, chloroquinone, and methyl gallate showed significant reduction in the case of DENV, while only methyl gallate showed a significant activity in the case of CHIKV. Earlier reports have shown that these compounds exert anti-dengue, antihelmintic, and antiparasitic effects (Hundt et al., 2015). Anacardic acid is obtained from *Anacardium occidentale* nuts. However, extracts prepared from *Anacardium occidentale* leaves did not show any antiviral activity, suggesting absence of active antiviral compounds in leaves. The present study suggests the use of methyl gallate as a standard for investigations of anti-chikungunya activity.

Plant extracts which show activity in pretreatment might modulate the host factors such as receptors to prevent virus entry and/or virus replication. The extracts which showed activity under cotreatment conditions might bind to the virus and prevent its binding to the cellular receptors for entry. The extracts which showed activity under posttreatment conditions might influence viral replication and/or assembly by interaction with viral proteins or host proteins that take part in these steps. Further studies are needed to find out the mechanism of action of the plant extracts.

In many of these experiments, though antiviral activity was visible in terms of reduction in infectious virus particles (FFU), a corresponding decrease in viral RNA titer was not observed. This suggests that the plant extracts which exerted complete reduction in terms of FFU but not viral RNA may not affect viral RNA replication but might inhibit the assembly of virus particles. Moreover, quantitative real-time RT-PCR is a more sensitive assay than FFU and detects RNA from even noninfectious particles also. Hence, minor differences in viral RNA titer may be not reflected in quantitative real-time RT-PCR assay results unless there is a major difference in the RNA titer. The extract which affected both FFU and viral RNA titer might inhibit viral RNA replication.

The concentration of the extracts which exerted antiviral activities was different from each other and it is possible that the extracts which had antiviral activity at lower concentrations still had high amount of the active antiviral compound while those extracts which exerted antiviral activity at higher concentration had lower amount of the active antiviral compounds. Those extracts which exerted antiviral activity at lower concertation may be further taken forward with whole formulation as a phytopharmaceutical drug while for the extracts which had antiviral activity at higher concentrations, there is a need to identify the active compound to be further considered an antiviral drug.

Identifying the active fractions and compounds from the extracts with anti-dengue and anti-chikungunya activities will help to develop the formulations based on the above plants as a phytopharmaceutical drug which can be further evaluated in preclinical and clinical studies. The present study paves the way for further focused research on plant based antivirals against DENV and CHIKV to find effective treatments against these debilitating viral diseases.

## Data Availability Statement

The original contributions presented in the study are included in the article/**Supplementary Material**. Further inquiries can be directed to the corresponding authors.

## Author Contributions

Conceived and designed the experiments: SC, DP, SH, and KA. Performed the experiments: PP, DC, MK, and MBK. Analyzed the data: DP, KA, PP, DC, MK, HH, MBK, and SC. Provided extracts: SH, RJ, HH, and MK. Wrote the paper: DP, KA, HH, RJ, SC, and SH. All authors listed have made a substantial, direct, and intellectual contribution to the work and approved it for publication.

## Funding

This work was supported by the ICMR-National Institute of Virology, Pune.

## Conflict of Interest

The authors declare that the research was conducted in the absence of any commercial or financial relationships that could be construed as a potential conflict of interest.

## Publisher’s Note

All claims expressed in this article are solely those of the authors and do not necessarily represent those of their affiliated organizations, or those of the publisher, the editors and the reviewers. Any product that may be evaluated in this article, or claim that may be made by its manufacturer, is not guaranteed or endorsed by the publisher.

## Supplementary Material

Supplementary Figure 1 Effect of different plant extracts and pure compounds on cell viability as measured by MTT assay. The results are expressed as mean percent cell viability ± standard error. The experiments were done in triplicates at two independent time points. Cytotoxic concentration that causes 50% cell death (CC50 values) were also provided.
