# Supplementary figures and images for: In Vitro Antiviral Activity of Potential Medicinal Plant Extracts Against Dengue and Chikungunya Viruses
Source: Front Cell Infect Microbiol. 2022 Apr 7;12:866452. doi: 10.3389/fcimb.2022.866452 (PMC9021897; doi:10.3389/fcimb.2022.866452)

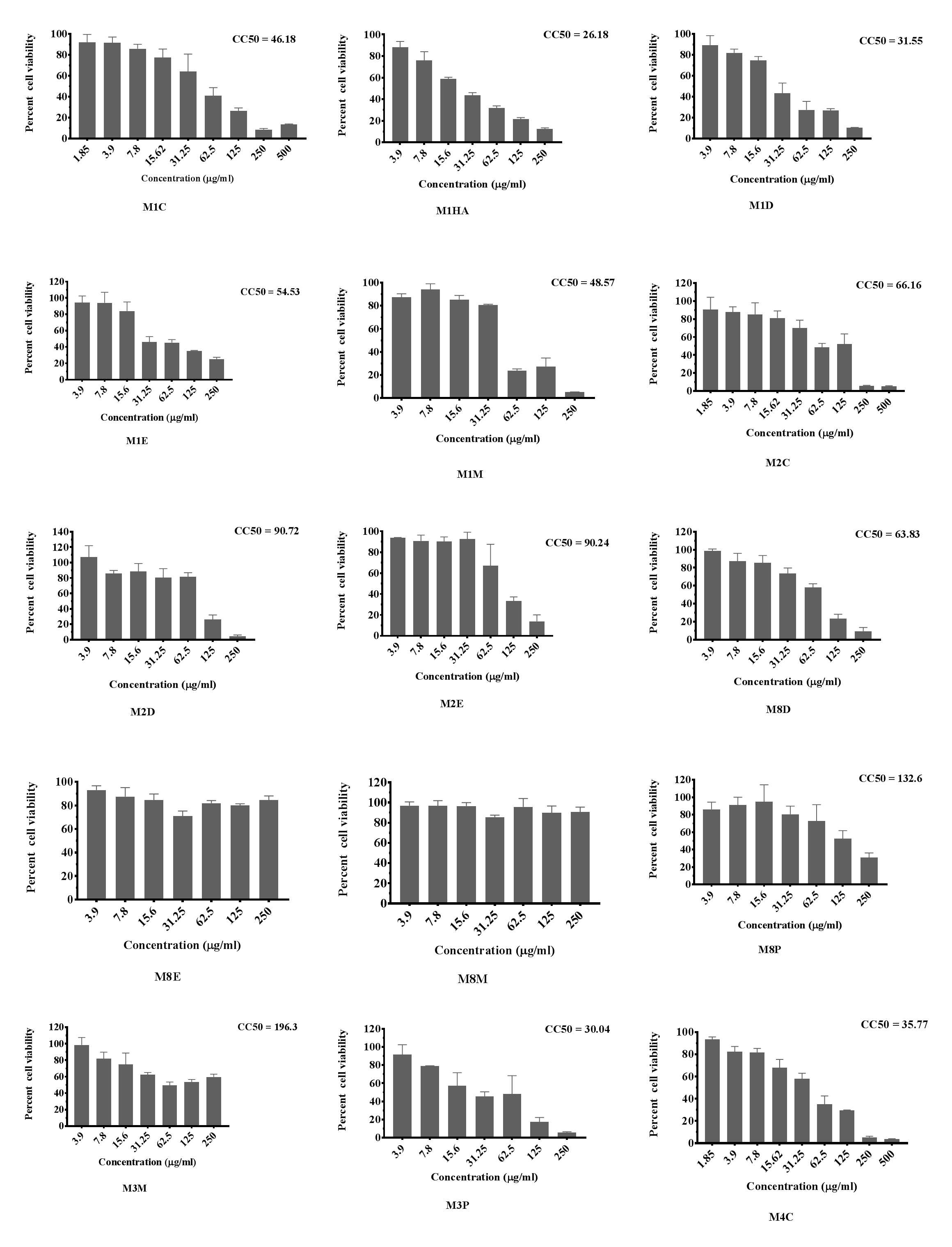

Supplement: Supplementary Figure 1 — Effect of different plant extracts and pure compounds on cell viability as measured by MTT assay. The results are expressed as mean percent cell viability ± standard error. The experiments were done in triplicates at two independent time points. Cytotoxic concentration that causes 50% cell death (CC50 values) were also provided. [file Image_1.tiff]
